# Delay-Induced Complexity and Chaotic Dynamics in a Network Model of Information Spreading

**DOI:** 10.3390/e28050570

**Published:** 2026-05-19

**Authors:** Vasyl Martsenyuk, Tomasz Gancarczyk

**Affiliations:** Department of Computer Science and Automatics, University of Bielsko-Biala, Willowa 2, 43-309 Bielsko-Biała, Poland; tgan@ubb.edu.pl

**Keywords:** information spreading, delay difference equations, nonlinear dynamics, stability analysis, bifurcation, chaotic dynamics, Lyapunov exponent, network models

## Abstract

Understanding how information spreads in complex networks is essential for analyzing social influence, opinion formation, and the emergence of collective behavior. In many real-world systems, interactions are not instantaneous but involve delays due to communication, cognition, and response times. Motivated by this observation, the present paper investigates a delayed network model of information spreading, focusing on how time delay and interaction strength shape the system’s dynamical behavior. The novelty of the proposed approach lies in the formulation of a discrete-time network model that explicitly incorporates delayed interactions within a nonlinear dynamical framework. Using delay difference equations, the model captures both local coupling effects and memory-driven feedback, allowing for a systematic study of their combined impact on stability and complexity. Analytical results establish the existence of steady states and provide conditions for their local stability, revealing critical thresholds at which the system undergoes qualitative transitions. These findings are complemented by extensive numerical simulations. In particular, bifurcation analysis and the computation of the largest Lyapunov exponent demonstrate a progression from stable equilibria to oscillatory behavior, and further to chaotic dynamics as the delay and coupling strength increase. Our results highlight the fundamental role of delay as a mechanism that enhances nonlinear complexity and promotes unpredictable dynamics in networked systems. These insights contribute to a deeper understanding of information propagation processes, and may inform the design and control of spreading phenomena in social and technological networks.

## 1. Introduction

The rapid dissemination of information in complex networks is a fundamental phenomenon underlying social media dynamics, opinion formation, and collective behavior. Mathematical modeling plays a crucial role in understanding how information spreads, persists, or vanishes across interconnected systems. In particular, network-based dynamical models provide a powerful framework for capturing interactions between agents and the mechanisms governing information flow [1,2,3].

In many real-world systems, information transmission is not instantaneous; time delays naturally arise due to cognitive processing, communication latency, and decision-making processes. Incorporating such delays into dynamical models significantly enriches their behavior, often leading to oscillations, bifurcations, and even chaotic dynamics. Consequently, delay difference and delay differential equations have become essential tools for studying complex temporal patterns in information propagation and related systems [4,5,6].

Motivated by these observations, this paper investigates a delayed network model of information spreading, focusing on the interplay between coupling strength and time delay. By combining analytical techniques with numerical simulations, we examine the system’s steady states, their stability properties, and the emergence of nonlinear phenomena. Special attention is given to the role of delay as a driving mechanism for complex dynamics, including transitions from stable equilibria to oscillatory and chaotic regimes. The proposed framework aims to bridge classical network-based diffusion models with nonlinear dynamical systems theory, providing new insight into how memory and interaction strength jointly shape collective behavior.

### 1.1. Related Works

**Delayed systems.** Time-delayed dynamical systems have emerged as a powerful framework for modeling memory and latency effects in complex processes, especially in biology, neural networks, epidemiology, control systems, and information propagation. The inclusion of delays captures the intrinsic lag between cause and effect, such as synaptic transmission in neuronal models or response times in social networks. For instance, delay differential equations have been used to model the spread of diseases with incubation periods [7], the dynamics of physiological regulation [4], and the stability of multi-agent consensus systems [5]. Neural and cognitive systems with delay feedback often exhibit complex dynamics, including oscillations and chaos [8]. Recent developments also focus on information diffusion over networks with delay-induced memory, enabling insights into social behaviors and collective decision-making [9].

**Lyapunov-based stability for delayed systems.** Recent research on the stability of delayed systems, particularly in the case of artificial neural networks modeled by delay differential equations, has centered around two main approaches. The first involves local analysis using linearization techniques, as seen in works like [10], where stability is determined via characteristic transcendental equations and Hopf bifurcation is explored using tools such as Rouché’s theorem, normal form theory, and the center manifold theorem. The second approach, which is more robust and flexible, uses Lyapunov–Krasovskii functionals to derive constructive stability conditions, often formulated as linear matrix inequalities (LMIs). This method not only allows for parameter optimization but also facilitates the estimation of exponential decay rates, making it suitable for complex systems. This includes systems with distributed, time-varying, or neutral-type delays, impulsive effects, or unbounded activation functions, as demonstrated in works such as [11,12].

**Nonlinear behavior and chaos investigation for dynamic models.** Studies on nonlinear behavior and chaos in dynamic models focus on understanding how nonlinearities, feedback mechanisms, and system parameters influence the emergence of complex and often unpredictable dynamics. Such investigations typically involve identifying bifurcations, that is, qualitative changes in system behavior, through analytical tools such as phase portrait analysis, Poincaré maps, and Lyapunov exponents, which help to quantify sensitivity to initial conditions [6,13]. In many physical, biological, and engineering systems, including neural networks, mechanical oscillators, and population dynamics, nonlinearities can give rise to periodic, quasi-periodic, and chaotic regimes [14]. Research in this area often combines theoretical analysis with numerical simulations in order to map stability regions, detect routes to chaos (e.g., period-doubling, intermittency, or quasi-periodicity), and explore control or synchronization strategies that either suppress or exploit chaotic behavior for applications such as secure communications or optimization [15]. Recent studies have further demonstrated that discrete nonlinear systems may exhibit rich dynamical phenomena, including hyperchaos, multistability, and coexisting attractors. For example, state-dependent variable fractional-order quadratic maps have been shown to generate hyperchaotic behavior suitable for secure information processing and image protection, while discrete memristive neuron maps reveal extreme multistability with implications for coupled dynamical systems and engineering applications [16,17]. These developments highlight the growing importance of nonlinear discrete-time frameworks for understanding complex emergent behavior.

**Recurrent neural network (RNN) modeling.** Modeling via RNNs focuses on representing and analyzing systems with feedback connections that allow internal states to evolve over time, making such models well suited for processing sequential or temporal data. Unlike feedforward architectures, RNNs incorporate memory of past inputs through recurrent links, enabling dynamic behavior and complex temporal dependencies. Mathematical modeling via RNNs often employs systems of nonlinear differential or difference equations, sometimes with time delays, in order to capture their continuous- or discrete-time evolution [12,18,19]. Research in this field addresses stability, convergence, and learning dynamics as well as phenomena such as oscillations, bifurcations, and chaos in network behavior [20]. Applications span signal processing, time-series prediction, pattern recognition, and control systems, with theoretical studies increasingly using tools such as Lyapunov methods, bifurcation theory, and linear matrix inequalities to design stable and efficient recurrent architectures [11].

**Graph-based models.** These models provide a powerful framework for representing and analyzing systems in which components and their relationships can be naturally described as nodes and edges, enabling the study of both structural and dynamic properties. These models are widely used across disciplines, including social network analysis [21], biological systems [22], communication networks [1], and machine learning [23]. In recent years, graph theory has been integrated with computational approaches such as graph neural networks (GNNs) to process complex and irregular data structures and to capture higher-order dependencies. Mathematical analysis of graph-based models often focuses on connectivity, centrality, clustering, spectral properties, and percolation phenomena, which influence processes such as diffusion, synchronization, and resilience to failures [1,24]. Advances in this area increasingly combine classical graph algorithms with probabilistic and optimization techniques, allowing for efficient modeling of large-scale, time-varying, and weighted graphs in practical applications ranging from recommendation systems to epidemic modeling [25].

**Random networks.** These networks are mathematical models in which nodes are connected according to probabilistic rules, providing a framework for studying the structure and dynamics of complex systems with inherent uncertainty. Originating from the classical Erdős–Rényi model, in which edges are formed independently with a fixed probability [26], the theory has expanded to include scale-free networks, small-world networks [25], and other variants that capture real-world features such as heavy-tailed degree distributions and high clustering [27]. Research in this area examines statistical properties such as degree distribution, connectivity, and robustness as well as dynamic processes on networks, including diffusion, synchronization, and epidemic spreading [2,28]. Analytical tools from probability theory, statistical physics, and spectral graph theory are often combined with numerical simulations in order to understand phase transitions, critical thresholds, and resilience under random failures or targeted attacks [29]. Applications span diverse domains where randomness plays a key role in shaping global behavior, including communication systems, biological networks, neuroscience, and social interactions [1,29].

**Modeling approaches to information spreading within social networks.** Such approaches aim to capture how ideas, opinions, or innovations propagate through interconnected individuals, drawing on methods from network science [25,30], epidemiology [31], and statistical physics [32,33]. Classical models such as the SI, SIR, and SIS frameworks adapt concepts from disease spreading to describe the adoption or decay of information [31,34], while threshold and cascade models account for social reinforcement effects in which individuals adopt information when exposure surpasses a personal or global threshold [35,36]. Recent advances incorporate heterogeneous network topologies, temporal dynamics, and multi-layer or multiplex structures in order to better reflect real-world social media platforms [37,38]. Analytical techniques, including mean-field approximations, percolation theory, and spectral analysis, are often complemented by agent-based simulations to explore factors such as community structure, influential spreaders, and competing or synergistic information streams [2,3]. Applications include predicting viral content, optimizing marketing strategies, and designing interventions to control misinformation or enhance beneficial information diffusion [39,40].

### 1.2. Main Contributions

The main contributions of this work can be summarized as follows:

- We propose a discrete-time network model of information spreading that explicitly incorporates time-delayed interactions and nonlinear response functions, allowing for the study of memory-driven effects in network dynamics.
- We derive analytical conditions for the existence and local stability of steady states, identifying critical parameter thresholds that govern qualitative changes in system behavior.
- We perform a comprehensive numerical investigation, including bifurcation analysis and computation of the largest Lyapunov exponent, revealing transitions between stable, oscillatory, and chaotic regimes.
- We demonstrate that time delay acts as a key mechanism driving nonlinear complexity, significantly enlarging regions of irregular and chaotic dynamics.
- We provide a unified interpretation of how coupling strength, attention decay, and delay jointly determine the global dynamical scenario of information spreading.

The remainder of the paper is organized as follows: Section 2 introduces the delayed network model and the analytical and numerical methods employed; Section 3 derives the equilibrium solutions and establishes their existence conditions; Section 4 investigates the local stability of equilibria and identifies critical thresholds for stability loss; Section 5 examines bifurcations, oscillatory behavior, and the emergence of chaotic dynamics using Lyapunov exponent analysis; Section 6 provides an overall interpretation of the system’s dynamical behavior, emphasizing the role of delay; finally, Section 7 summarizes the main results and outlines directions for future research.

## 2. Model Formulation

In this section, we introduce the mathematical framework used to model the spreading of information within a social network. The model captures key mechanisms observed in real social systems, including interpersonal information transmission, temporal delays in user responses, and gradual loss of interest or memory. The formulation is discrete in time and is designed to accommodate dynamic network structures and heterogeneous user behavior.

### 2.1. Network Structure and Generation

The social interaction network is modeled as a directed graph

(1) G(t)=(V,E(t)),

where V={1,…,N} denotes the set of users (agents) participating in the information exchange process and E(t) represents the set of directed communication links at time *t*. A directed edge from node *j* to node *i* indicates that user *j* can transmit information to user *i*.

The connectivity structure is encoded by the (possibly time-dependent) adjacency matrix

(2) $$A\left(t\right)=\left(a_{ij}\left(t\right)\right)\in {\left\{0,1\right\}}^{N\times N},$$

where

(3) $$a_{ij}\left(t\right)=\left\{\begin{array}{cc} 1, & \mathrm{if}\;a\;\mathrm{directed}\;\mathrm{connection}\;\mathrm{from}\;\mathrm{node}\;j\;\mathrm{to}\;\mathrm{node}\;i\;\mathrm{exists}\;\mathrm{at}\;\mathrm{time}\;t,\\ 0, & \mathrm{otherwise}. \end{array}\right.$$

This time-varying formulation allows the model to capture temporal changes in user activity, communication patterns, and network topology. However, for the numerical simulations presented in this work, the network topology is assumed to remain fixed after generation, i.e., A(t)≡A.

In this study, the underlying network is generated using the Erdős–Rényi random graph model [41,42], where each possible directed edge is included independently with a fixed probability p∈(0,1). This construction yields a homogeneous random graph with binomial (or asymptotically Poisson) degree distribution, providing a mathematically tractable framework for analyzing dynamical processes on networks.

The use of the Erdős–Rényi model is motivated by its simplicity and its widespread adoption as a baseline in the study of complex networked systems. In particular, it enables us to isolate and investigate the intrinsic dynamical effects induced by nonlinear interactions and time delays without introducing additional structural heterogeneities.

Nevertheless, real-world social networks typically exhibit structural features not captured by the Erdős–Rényi model, including high clustering, community organization, and heavy-tailed degree distributions. These characteristics may substantially influence information spreading dynamics. Accordingly, the results presented herein should be interpreted as revealing fundamental dynamical mechanisms rather than as providing a direct quantitative representation of empirical social systems.

Finally, the nonlinear interactions between connected nodes are mediated through activation functions, choice of which plays a crucial role in determining the overall system dynamics. The role of activation functions is discussed in detail in the subsequent sections.

### 2.2. Information Awareness Dynamics

Each user i∈V is characterized by an awareness level $x_{i}\left(t\right)$ at discrete time *t*, which represents the degree to which the user is informed or engaged with a specific topic. Negative awareness values can be interpreted as opposition, skepticism, or counter-information influence within the network. The temporal evolution of awareness is governed by a *discrete-time information spreading model*

(4) $$x_{i}\left(t+1\right)=\sigma (\left(1-\alpha_{i}\left(t\right)\right)x_{i}\left(t\right)+\beta \sum_{j\in V}a_{ji}\left(t\right)p_{ji}\left(t-\tau_{j}\right)x_{j}\left(t-\tau_{j}\right)),$$

where the first term inside the activation function models the persistence of the user’s current awareness level, reduced by a time-dependent forgetting rate $\alpha_{i}\left(t\right)$. This parameter captures the natural tendency of users to lose interest or forget information over time, and may implicitly reflect effects such as topic saturation or information overload within the network.

The second term represents the influence exerted by neighboring users. The propagation rate β controls the overall strength of information transmission across the network and represents a forgetting (or decay) rate, commonly used in information diffusion and neural dynamics models [43], while the adjacency matrix element $a_{ji}\left(t\right)$ ensures that only active communication links contribute to the update. The factor $p_{ji}\left(t-\tau_{j}\right)$ denotes the probability that user *j* successfully influences user *i* after a delay $\tau_{j}$, accounting for individual response times and asynchronous interactions. The presence of the delay term $x_{j}\left(t-\tau_{j}\right)$ allows the model to capture realistic temporal lags between information exposure and its effect on awareness.

The nonlinear activation function σ(·) is chosen as the hyperbolic tangent, which bounds the awareness level and introduces saturation effects. Alternative activation functions could also be considered (e.g., ReLU, piecewise-linear functions), but tanh provides smoothness and boundedness, which are essential for the applied Lyapunov analysis. This nonlinearity reflects cognitive limitations in information processing and prevents unbounded growth of awareness, ensuring that the system remains within a realistic dynamical range. Together, these components form a flexible framework for studying delayed information spreading dynamics in time-varying social networks.

### 2.3. Forgetting Model

To represent the decay of user memory and sustained engagement over time, we introduce a time-dependent forgetting model that combines intrinsic memory loss with reinforcement induced by social interactions. The forgetting rate of user *i* at time *t* is defined as

(5) $$\alpha_{i}\left(t\right)=\alpha_{0}e^{-\lambda t}-\gamma \sum_{j\in V}p_{ji}\left(t\right)x_{j}\left(t\right),$$

where $\alpha_{0}$ denotes the initial forgetting rate, reflecting the baseline tendency of users to lose awareness in the absence of external stimuli. The exponential decay term governed by λ captures the gradual temporal evolution of forgetting, modeling the reduction in attention or memory retention as the novelty of information diminishes.

The second term accounts for social reinforcement effects arising from repeated exposure to information through interactions with other users. The γ parameter controls the strength of this reinforcement mechanism, while the summation aggregates the influence of neighboring users weighted by their awareness levels and influence probabilities. As users receive repeated signals from their social environment, the effective forgetting rate is reduced, allowing awareness to persist for longer periods. This formulation enables the forgetting process to adapt dynamically to the evolving state of the network and the intensity of information exchange.

### 2.4. Model Parameters and Assumptions

The model is governed by a set of parameters that regulate information propagation, memory decay, and social reinforcement. The propagation rate β>0 determines the overall strength of awareness transmission through active network connections. Larger values of β correspond to faster diffusion processes, while smaller values result in slower propagation and may lead to the eventual disappearance of awareness. In the extended analysis, β is treated as a generalized decay parameter, allowing for exploration beyond strict probabilistic interpretation.

The forgetting dynamics are controlled by the parameters $\alpha_{0}$, λ, and γ. The parameter $\alpha_{0}\in \left(0,1\right)$ represents the initial forgetting rate, while λ>0 governs the temporal evolution of this baseline decay. The reinforcement parameter γ≥0 quantifies the extent to which repeated social interactions mitigate forgetting and sustain awareness.

User influence is captured by the probability function $p_{ji}\left(t\right)$, which represents the likelihood of information from user *j* affecting user *i* at time *t*. This function is assumed to be bounded in the interval [0,1], and may depend on interaction frequency, tie strength, or other contextual factors. Time delays $\tau_{j}\geq 0$ are introduced to account for heterogeneous response times and non-instantaneous information processing.

The awareness variable $x_{i}\left(t\right)$ is assumed to be continuous and bounded due to the use of the hyperbolic tangent activation function. This choice introduces saturation effects and ensures that awareness levels remain within a realistic range. Unless otherwise specified, parameters are assumed to be homogeneous across users, although the framework readily accommodates heterogeneous extensions for empirical applications.

Collectively, these assumptions define a coherent and analytically tractable framework for investigating delayed information spreading processes in dynamic social networks.

The modeling framework introduced above provides a unified description of information spreading dynamics that incorporates time-varying network structure, delayed interactions, and adaptive forgetting mechanisms. While numerical simulations offer valuable insight into the qualitative behavior of the system, a rigorous understanding of its long-term dynamics requires analytical investigation. In particular, it is essential to characterize the conditions under which awareness levels converge to steady states, persist over time, or exhibit complex dynamical behavior.

Equation (4) can be viewed as a discrete-time information spreading model that is related to classical opinion dynamics such as the DeGroot model [44] and bounded confidence models [45] as well as to nonlinear network dynamics inspired by neural systems [46,47]. From a network science perspective, it is also connected to models of information diffusion [48] and epidemic spreading processes [31]. The inclusion of time delay is motivated by studies on delayed interactions in complex networks [49,50].

The forgetting rate (5) is modeled as an exponential decay term, consistent with classical memory decay theory [51] and recent information diffusion models incorporating forgetting mechanisms [52,53].

The following section focuses on the stability properties of the proposed model. By analyzing the existence and stability of equilibrium points, we derive sufficient conditions for the decay or persistence of information within the network. Our analysis highlights the roles played by propagation strength, forgetting rates, reinforcement effects, and transmission delays in shaping the asymptotic behavior of the system.

## 3. Steady States

In this section, we investigate the steady-state behavior of the proposed awareness dynamics. The analysis focuses on the existence and characterization of equilibrium solutions under both simplified analytical settings and more general network configurations. These results provide insight into the long-term outcomes of information spreading processes governed by delayed interactions and adaptive forgetting.

To understand the asymptotic behavior of the awareness model, we analyze its steady states $x^{⋆}=\left(x_{1}^{⋆},x_{2}^{⋆},\ldots ,x_{N}^{⋆}\right)$, defined as fixed points satisfying $x_{i}\left(t+1\right)=x_{i}\left(t\right)=x_{i}^{⋆}$ as t→∞. At steady state, the general awareness update equation reduces to

(6) $$x_{i}\left(t+1\right)=\sigma \left(\left(1-\alpha_{i}\right)x_{i}\left(t\right)+\beta \sum_{j\in V}p_{ji}\left(t-\tau_{j}\right)x_{j}\left(t-\tau_{j}\right)\right),$$

where σ(·) denotes a nonlinear activation function such as the hyperbolic tangent or sigmoid.

The choice of activation function plays a fundamental role in determining the qualitative behavior of the proposed information spreading model. In the simplified analysis, a sigmoid activation function is employed due to its monotonicity and straightforward interpretation in terms of bounded awareness levels. Since the sigmoid maps its input to the interval (0,1), it is naturally suited for modeling non-negative awareness states or information adoption probabilities, where 0 corresponds to complete unawareness and values close to 1 indicate maximal awareness or saturation. Under this formulation, the spreading process is interpreted as purely accumulative, involving only non-negative influence and reinforcement of information.

In the general formulation, the hyperbolic tangent activation function is adopted due to its symmetry and analytical convenience, particularly for stability analysis. Unlike the sigmoid function, tanh maps its input to the interval (−1,1), allowing the model to represent both positive and negative awareness states. This extended range enables the incorporation of antagonistic or oppositional reactions to information, where negative values may be interpreted as skepticism, rejection, counter-opinion, or resistance to the spreading content. Such a representation is particularly relevant for social systems in which information dissemination may generate polarized responses rather than uniform acceptance.

From a mathematical perspective, the hyperbolic tangent function also offers important analytical advantages due to its odd symmetry, smoothness, and bounded derivative, which facilitate the derivation of stability conditions and bifurcation thresholds. Although other bounded nonlinear activation functions could be considered within the proposed framework, the specific quantitative location of stability boundaries and bifurcation points may depend on the chosen functional form.

Assuming time-invariant dynamics, absence of external inputs, and convergence to equilibrium, the delayed system yields a system of nonlinear algebraic equations of the form

(7) $$x_{i}^{⋆}=\sigma \left(\left(1-\alpha_{i}^{⋆}\right)x_{i}^{⋆}+\beta \sum_{j\in V}p_{ji}^{⋆}x_{j}^{⋆}\right),$$

where $\alpha_{i}^{⋆}=\lim_{t\to \infty }\alpha_{i}\left(t\right)$ and $p_{ji}^{⋆}=\lim_{t\to \infty }p_{ji}\left(t\right)$ for all i,j∈{1,…,N}. These equations define the possible equilibrium awareness levels supported by the network and the model parameters.

It is important to note that the admissibility of trivial steady states depends on the choice of activation function. In particular, for sigmoid activation functions, the zero-awareness state $x^{⋆}\equiv 0$ is not a solution, since σ(0)≠0. For this reason, the hyperbolic tangent activation function is adopted in the subsequent analysis, as it permits the existence of the trivial equilibrium and facilitates analytical tractability.

### 3.1. Analytical Solution in a Simplified Symmetric Case

To gain analytical insight, we first consider a simplified symmetric setting consisting of two mutually interacting users. In this case, the steady-state awareness levels satisfy $x_{1}^{⋆}=x_{2}^{⋆}=x^{⋆}$, the forgetting rate is assumed constant with value α, and the mutual influence probabilities are equal, $p_{12}=p_{21}=p$. Under these assumptions, the steady-state equation reduces to

(8) $$x^{⋆}=\sigma \left(\left(1-\alpha +\beta p\right)x^{⋆}\right).$$

Introducing the effective coupling parameter c=1−α+βp, the fixed-point condition becomes a transcendental equation. For sigmoid activation, this equation takes the form

(9) $$x^{⋆}=\frac{1}{1+e^{-cx^{⋆}}},$$

which generally admits no closed-form solution and must be solved numerically. However, for small values of *c*, a first-order Taylor approximation of the sigmoid function yields

(10) $$x^{⋆}\approx \frac{1}{2}+\frac{cx^{⋆}}{4},$$

leading to the approximate solution

(11) $$x^{⋆}\approx \frac{1/2}{1-c/4}.$$

This expression highlights the explicit dependence of the steady-state awareness level on the balance between forgetting and reinforcement effects, as captured by the parameter *c*.

### 3.2. Numerical Solution in General Erdős–Rényi Networks

For more realistic network configurations, analytical solutions are generally intractable, and numerical methods are employed to compute steady states. We consider directed Erdős–Rényi random graphs G(N,p) and construct a column-normalized influence matrix $P=[p_{ji}]$. In vector form, the steady-state condition can be written as

(12) $$x^{⋆}=\sigma \left(\left(I-A\right)x^{⋆}+\beta Px^{⋆}\right),$$

where $A=\mathrm{diag}(\alpha_{1},\ldots ,\alpha_{N})$ is the diagonal matrix of forgetting rates.

The steady-state solution is obtained using an iterative fixed-point scheme defined by

(13) $$x^{\left(k+1\right)}=\sigma \left(\left(I-A\right)x^{\left(k\right)}+\beta Px^{\left(k\right)}\right),$$

which is initialized with a non-negative awareness vector and repeated until convergence, measured by $∥x^{\left(k+1\right)}-x^{\left(k\right)}∥<\varepsilon$ for a prescribed tolerance ε. Due to the boundedness and smoothness of the hyperbolic tangent activation function, the iteration exhibits stable convergence behavior across a wide range of parameter values.

The resulting steady-state solutions reveal how network connectivity, influence heterogeneity, and forgetting rates jointly shape long-term awareness distributions. In particular, highly connected nodes and strongly reinforced subgraphs tend to sustain elevated awareness levels, while sparsely connected regions experience faster decay.

## 4. Stability Analysis

In this section, we investigate the stability properties of the proposed discrete-time information spreading model in the presence of discrete delays. The analysis is carried out using a Lyapunov–Krasovskii functional approach, which provides sufficient conditions for global asymptotic and exponential stability of the trivial equilibrium. The results highlight how forgetting rates, coupling strength, activation nonlinearity, and transmission delays jointly affect the long-term behavior of the system.

**Lyapunov–Krasovskii Functional for Discrete-Time System with Discrete Delays**

We consider the discrete-time information spreading model in (4). We propose the following Lyapunov–Krasovskii functional to study the stability of the steady state of (4).

**Sector condition on activation function:** We assume that $g\left(x\right)={\left[g_{1}\left(x_{1}\right),\ldots ,g_{n}\left(x_{n}\right)\right]}^{⊤}$ satisfies the sector condition:

(14) $$g_{j}\left(0\right)=0,\;0\leq \frac{g_{j}\left(\xi_{1}\right)-g_{j}\left(\xi_{2}\right)}{\xi_{1}-\xi_{2}}\leq l_{j},\;\forall \xi_{1}\neq \xi_{2},\;l_{j}>0$$

and define the diagonal matrix $L=\mathrm{diag}(l_{1},l_{2},\ldots ,l_{n})$.

**Proposed Lyapunov functional:**

(15) $$V\left[t\right]=\rho^{t}x^{⊤}\left(t\right)Px\left(t\right)+\sum_{j\in V}\rho^{t-\tau_{j}}g^{⊤}\left(x_{j}\left(t-\tau_{j}\right)\right)Q_{j}g\left(x_{j}\left(t-\tau_{j}\right)\right),$$

where:

- ρ>1 is a decay factor
- $P\in R^{n\times n}$ is symmetric and positive definite
- $Q_{j}\in R^{n\times n}$ are symmetric and positive-definite weighting matrices for each delayed input
- $\tau_{j}\in N$ is the delay associated with node *j*.

The first term $V_{1}\left[t\right]=\rho^{t}x^{⊤}\left(t\right)Px\left(t\right)$ captures the present-state energy with exponential decay. The second term $V_{2}\left[t\right]$ accounts for the contribution of delayed activations.

This functional structure mirrors the continuous-time Lyapunov functional with discrete delays, but is tailored to the discrete-time domain.

To analyze stability, it is necessary to estimate the difference ΔV[t]=V[t+1]−V[t] along trajectories of (4), using the sector condition in (14) and bounding the nonlinear terms via matrix inequalities.

**Step 1: Compute the Difference** ΔV[t]. We evaluate the one-step difference of V[t]:

(16) $$\begin{array}{cc} \Delta V[t] & =V[t+1]-V[t]\\ \; & =\rho^{t+1}x^{⊤}\left(t+1\right)Px\left(t+1\right)-\rho^{t}x^{⊤}\left(t\right)Px\left(t\right)\\ \; & \;+\sum_{j\in V}\left[\rho^{t+1-\tau_{j}}g^{⊤}\left(x_{j}\left(t+1-\tau_{j}\right)\right)Q_{j}g\left(x_{j}\left(t+1-\tau_{j}\right)\right)\right.\\ \; & \;\left.-\rho^{t-\tau_{j}}g^{⊤}\left(x_{j}\left(t-\tau_{j}\right)\right)Q_{j}g\left(x_{j}\left(t-\tau_{j}\right)\right)\right]. \end{array}$$

- **Step 2: Use the Sector Condition.** Define

(17) $$z_{i}\left(t\right):=\left(1-\alpha_{i}\left(t\right)\right)x_{i}\left(t\right)+\beta \sum_{j\in V}a_{\mathrm{ji}}\left(t\right)p_{\mathrm{ji}}\left(t-\tau_{j}\right)x_{j}\left(t-\tau_{j}\right)$$

so that $x_{i}\left(t+1\right)=g_{i}\left(z_{i}\left(t\right)\right)$.

Let $z\left(t\right)=M\left(t\right)x\left(t\right)+\beta A\left(t\right)P\left(t\right)x_{d}\left(t\right)$, where:

- $M\left(t\right)=\mathrm{diag}(1-\alpha_{i}\left(t\right))$
- $A\left(t\right)=[a_{\mathrm{ji}}\left(t\right)]$
- $P\left(t\right)=[p_{\mathrm{ji}}\left(t-\tau_{j}\right)]$
- $x_{d}\left(t\right)$ is the stacked vector $\left[x_{j}\left(t-\tau_{j}\right)\right]$.

Then,

(18) $$x\left(t+1\right)=g\left(z\left(t\right)\right)\;\mathrm{and}\;x^{⊤}\left(t+1\right)\mathrm{Px}\left(t+1\right)\leq z^{⊤}\left(t\right)\mathrm{LPLz}\left(t\right).$$

Thus, the first term in ΔV[t] can be estimated as

(19) $$\begin{array}{cc} \rho^{t+1}x^{⊤}\left(t+1\right)Px\left(t+1\right) & \leq \rho^{t+1}z^{⊤}\left(t\right)LPLz\left(t\right)\\ \; & =\rho^{t+1}\left[x^{⊤}M^{⊤}LPLMx+2\beta x^{⊤}M^{⊤}LPLAPx_{d}\right.\\ \; & \;\left.+\beta^{2}x_{d}^{⊤}P^{⊤}A^{⊤}LPLAPx_{d}\right]. \end{array}$$

**Step 3: Estimate Delay Terms.** For the second term in (16), we use a loose upper bound

(20) $$\Delta V_{2}\left[t\right]\leq \sum_{j\in V}\rho^{t+1-\tau_{j}}g^{⊤}\left(x_{j}\left(t+1-\tau_{j}\right)\right)Q_{j}g\left(x_{j}\left(t+1-\tau_{j}\right)\right).$$

Applying the sector condition

(21) $$g^{⊤}\left(x_{j}\left(\cdot \right)\right)Q_{j}g\left(x_{j}\left(\cdot \right)\right)\leq x_{j}^{⊤}\left(\cdot \right){\mathrm{LQ}}_{j}{\mathrm{Lx}}_{j}\left(\cdot \right),$$

we obtain a bound in terms of $x_{d}\left(t\right)$.

**Step 4: Matrix Formulation.** Define the following stacked vector:

(22) $$\xi \left(t\right):=\left[\begin{array}{c} x(t)\\ x_{d}\left(t\right) \end{array}\right].$$

The difference ΔV[t] is bounded as

(23) $$\Delta V\left[t\right]\leq \xi^{⊤}\left(t\right)\Gamma \xi \left(t\right)$$

with

(24) $$\Gamma =\left[\begin{array}{cc} \Gamma_{11} & \Gamma_{12}\\ \Gamma_{12}^{⊤} & \Gamma_{22} \end{array}\right],$$

where:

$$\begin{array}{cc} \Gamma_{11} & =\rho M^{⊤}LPLM-P\\ \Gamma_{12} & =\rho \beta M^{⊤}LPLAP\\ \Gamma_{22} & =\rho \beta^{2}P^{⊤}A^{⊤}LPLAP+\sum_{j\in V}\rho^{1-\tau_{j}}LQ_{j}L. \end{array}$$

**Stability Condition.**  *If Γ≺0, then ΔV[t]<0, and the zero solution of the system is globally asymptotically stable.*

**Theorem** **1**  (Stability Condition). *Assume that the activation function g(·) satisfies the sector condition. If there exists ρ>1 and symmetric positive definite matrices P, $Q_{j}$ such that*

(25) $$\Gamma =\left[\begin{array}{cc} \rho M^{⊤}LPLM-P & \rho \beta M^{⊤}LPLAP\\ {}* & \rho \beta^{2}P^{⊤}A^{⊤}LPLAP+\sum_{j\in V}\rho^{1-\tau_{j}}LQ_{j}L \end{array}\right]≺0,$$

*then the trivial solution of the system is globally asymptotically stable.*

### 4.1. Exponential Estimate

**Theorem** **2**  (Exponential Stability Estimate). *Let the activation function be g(x)=tanh(x) and suppose that there exist a scalar ρ>1 and symmetric positive-definite matrices P, $Q_{j}$ such that the matrix*

(26) $$\Gamma =\left[\begin{array}{cc} \rho M^{⊤}PM-P & \rho \beta M^{⊤}PAP\\ {}* & \rho \beta^{2}P^{⊤}A^{⊤}PAP+\sum_{j\in V}\rho^{1-\tau_{j}}Q_{j} \end{array}\right]≺0.$$

*Then, the zero solution of the system*

(27) $$x_{i}\left(t+1\right)=\tanh\left(\left(1-\alpha_{i}\left(t\right)\right)x_{i}\left(t\right)+\beta \sum_{j\in V}a_{\mathrm{ji}}\left(t\right)p_{\mathrm{ji}}\left(t-\tau_{j}\right)x_{j}\left(t-\tau_{j}\right)\right)$$

*is globally exponentially stable. Moreover, the solution satisfies the estimate*

(28) $$∥x\left(t\right)∥\leq C\cdot \kappa^{t}\cdot {∥\phi ∥}_{C},\;\mathrm{with}\;\kappa =\rho^{-1/2}\in \left(0,1\right),$$

*where C>0 is a constant depending on P and where ${∥\phi ∥}_{C}:=\sup_{s\in [-\tau_{\max},0]}∥x\left(s\right)∥$ is the norm of the initial function.*

**Proof.**  Define the Lyapunov–Krasovskii functional:

(29) $$V\left[t\right]=\rho^{t}x^{⊤}\left(t\right)\mathrm{Px}\left(t\right)+\sum_{j\in V}\rho^{t-\tau_{j}}g^{⊤}\left(x_{j}\left(t-\tau_{j}\right)\right)Q_{j}g\left(x_{j}\left(t-\tau_{j}\right)\right).$$

Under the given condition Γ≺0, it can be shown that $\Delta V\left[t\right]:=V\left[t+1\right]-V\left[t\right]\leq -{\delta ∥x\left(t\right)∥}^{2}$ for some δ>0. Since P≻0 and g(x)=tanh(x) satisfies the sector condition with bound $L=I_{n}$, it follows that

(30) $$V\left[t\right]\geq \rho^{t}\lambda_{\min}{\left(P\right)∥x\left(t\right)∥}^{2}\Rightarrow {∥x\left(t\right)∥}^{2}\leq \frac{V[0]}{\lambda_{\min}\left(P\right)}\cdot \rho^{-t}.$$

Setting $\kappa :=\rho^{-1/2}$ and $C:=\sqrt{V\left[0\right]/\lambda_{\min}\left(P\right)}$, the result follows. □

### 4.2. Exponential Stability Estimate: Numerical Verification

To validate the theoretical stability condition derived in Theorem 1, we consider the discrete-time delayed information spreading model (4), where g(·)=tanh(·), $\alpha_{i}>0$ is the self-decay coefficient, β is the coupling strength, and $\tau_{j}$ are constant discrete delays. The Erdős–Rényi network is taken as a fully-connected network without self-loops, i.e.,

(31) $$A=1_{n\times n}-I_{n},\;p_{\mathrm{ji}}=a_{\mathrm{ji}},$$

and the parameters are chosen as follows:

(32) $$n=37,\;\beta =0.01,\;\rho =1.5,\;T=40,\;\tau_{j}\equiv 5,\;\alpha_{i}\equiv 0.5.$$

This corresponds to M=diag(0.5), $\tau_{\max}=5$, and $Q_{j}=0.5I_{n}$ for all j.

The Lyapunov–Krasovskii functional is taken in the form

(33) $$V\left[t\right]=\rho^{t}x^{⊤}\left(t\right)\mathrm{Px}\left(t\right)+\sum_{j=1}^{n}\rho^{t-\tau_{j}}g^{⊤}\left(x_{j}\left(t-\tau_{j}\right)\right)Q_{j}g\left(x_{j}\left(t-\tau_{j}\right)\right),$$

where P≻0, $Q_{j}≻0$. Using the discrete Schur complement condition, we construct the block matrix

(34) $$\Gamma =\left[\begin{array}{cc} \rho M^{⊤}PM-P & \rho \beta M^{⊤}PA\\ {}* & -\sum_{j=1}^{n}\rho^{1-\tau_{j}}Q_{j} \end{array}\right]≺0.$$

The LMI conditions were solved using the CVXPY package (version 1.8), a Python(version 3.12.7)-based convex optimization framework [54], with warm-start initialization $P\left(0\right)=0.1I_{n}$, yielding

(35) $$\min\lambda \left(P\right)\approx 1.74\times 10^{-6},\;\max\lambda \left(P\right)\approx 1.93\times 10^{-6},$$

(36) minλ(Γ)≈−3.654.

From the definition of V[0] and the bound $\lambda_{\min}\left(P\right)$, the exponential estimate

(37) $$∥x\left(t\right)∥\leq C\kappa^{t},\;\kappa =\rho^{-1/2}\approx 0.8165$$

is obtained with C≈1.8755. Therefore, the explicit bound is

(38) $$∥x\left(t\right)∥\leq 1.8755\cdot {\left(0.8165\right)}^{t},\;t\geq 0.$$

**Figure 1:**
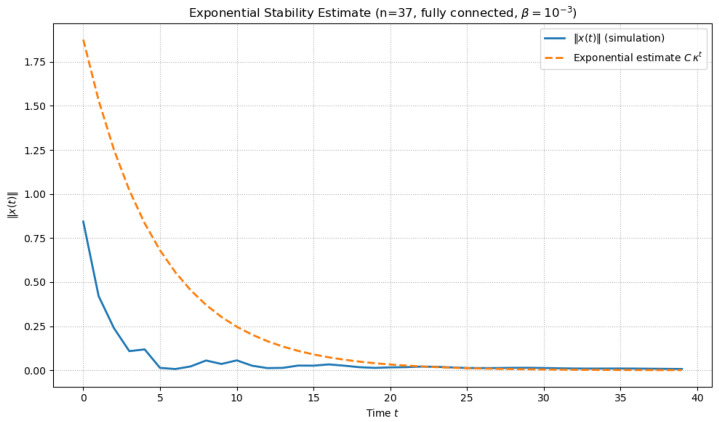
Exponential estimate $∥x\left(t\right)∥\leq C\kappa^{t}$ for the chosen parameters.

Figure 1 shows the computed exponential estimate.

In the simulation, the observed trajectory norm ∥x(t)∥ decays faster than the theoretical bound, confirming that the LMI-based condition produces a conservative but valid exponential estimate for the system in (4).

## 5. Nonlinear Dynamics and Complexity Analysis

### 5.1. Bifurcation Analysis

#### 5.1.1. Bifurcation Analysis with Respect to the Coupling Strength β and Attention–Decay Parameter α

To explore the nonlinear dynamics of the delayed network model, we construct bifurcation diagrams with respect to two key control parameters: the coupling strength β, and the attention–decay parameter α. For each fixed value of the delay τ, the asymptotic values of a representative node $x_{i}\left(t\right)$ are recorded after discarding transient dynamics. The activation function is chosen as g(x)=tanh(x), ensuring bounded trajectories in (−1,1).

**Figure 2:**
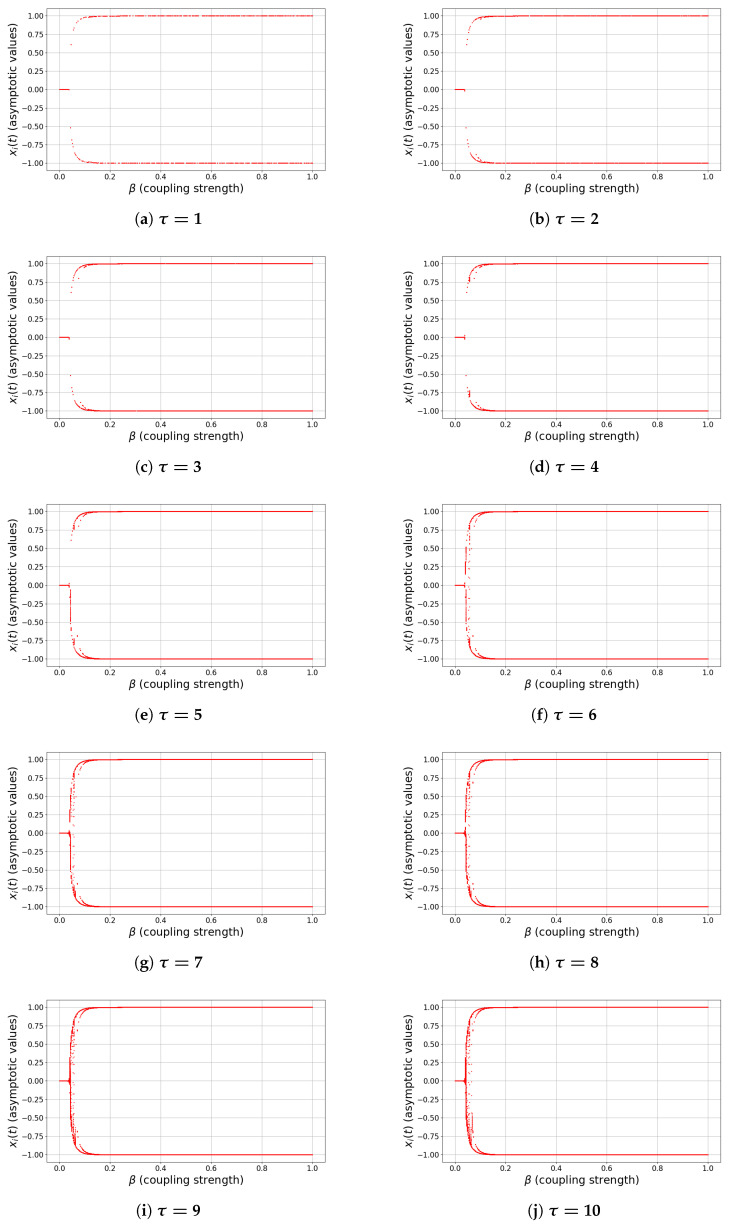
Bifurcation diagrams with respect to β for different delay values τ=1,…,10.

**Figure 3:**
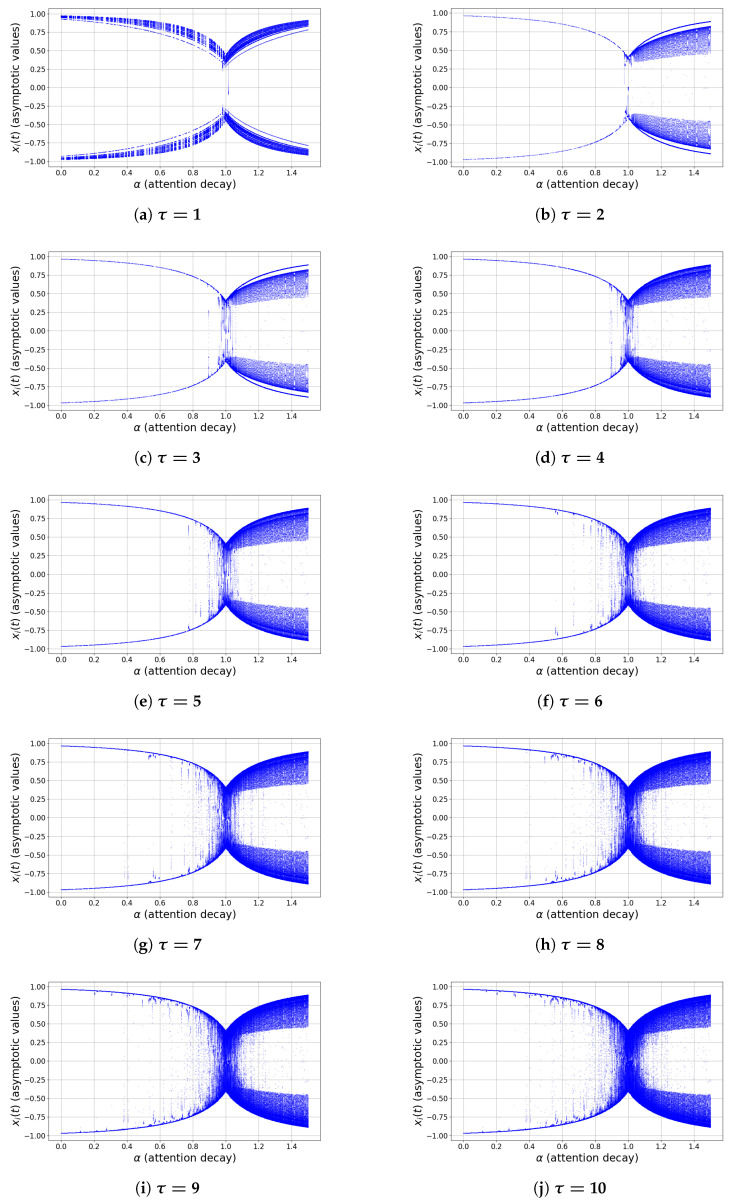
Bifurcation diagrams with respect to the coupling parameter α for increasing delay values τ=1,…,10. The panels demonstrate the progressive effect of delay on the bifurcation structure and the emergence of chaotic dynamics in the proposed information spreading model.

Figure 2a–j illustrates the bifurcation structure with respect to β, while Figure 3a–j presents the corresponding diagrams for α. Despite the different roles of these parameters, both induce qualitatively similar dynamical transitions, revealing a unified scenario governed by the balance between self-dynamics, network interaction, and delay.

(i) Weak interaction regime (small β, small α)

For sufficiently small coupling strength β or small attention decay α, the system exhibits stable and simple dynamics. In the case of small β, the trivial equilibrium $x_{i}\left(t\right)\equiv 0$ is globally attracting (Figure 2a–j). For small α, the effective self-weight (1−α) remains close to unity, leading to rapid convergence toward polarized states $x_{i}\left(t\right)\approx \pm 1$ (Figure 3a–d). In both cases, the dynamics are dominated by local effects and exhibit low complexity.

(ii) Symmetry-breaking and transition regime

As β increases beyond a critical threshold $\beta_{c}\left(\tau \right)$, the trivial equilibrium loses stability and symmetric branches emerge near x=±1, indicating bistability and consensus formation. Similarly, as α approaches the critical value α≈1, the effective self-feedback (1−α) weakens and the system transitions away from stable polarized states. In both cases, the bifurcation diagrams show branching structures and the coexistence of multiple attractors, reflecting increased sensitivity to initial conditions.

(iii) Oscillatory and complex dynamics

For intermediate values of β and in the vicinity of α≈1, the bifurcation diagrams exhibit dense vertical spreads and multiple overlapping branches (Figure 2d–i and Figure 3e–i). These features indicate the emergence of multi-periodic oscillations and irregular dynamics. In some cases, structures reminiscent of period-doubling cascades can be observed. This regime corresponds to the onset of complex dynamics, where delayed interactions and nonlinear coupling jointly increase the effective dimensionality of the system.

(iv) Saturation and strong nonlinearity regime

For sufficiently large β, the system converges to saturated states $x_{i}\left(t\right)\approx \pm 1$, resulting in two dominant horizontal branches in the bifurcation diagrams. Similarly, for α>1, the sign change in (1−α) introduces an effective inversion of self-feedback, but the bounded nonlinearity eventually stabilizes the system in large-amplitude states. In both cases, strong nonlinearity suppresses oscillations and restores regular behavior.

#### 5.1.2. Influence of Delay

The delay parameter τ plays a crucial and unifying role across both types of bifurcation scenarios. For small delays (e.g., τ=1,2,3), the transition between regimes is relatively sharp and the region of complex dynamics is narrow. As τ increases (Figure 2f–j and Figure 3f–j), the intermediate regime expands significantly and the bifurcation structure becomes increasingly intricate; in particular, the density of asymptotic states increases and extended regions of irregular behavior appear. This confirms the classical role of delay as a destabilizing mechanism, promoting oscillations and amplifying nonlinear effects before eventual saturation restores stability.

In summary, both parameters β and α induce a common sequence of dynamical transitions:

(39) simplestablebehavior⟶symmetrybreaking/transition

(40) ⟶complexoscillatorydynamics⟶saturatedstates.

The extent and complexity of the intermediate regime are strongly controlled by the delay τ, which acts as the primary source of dynamical richness in the system.

### 5.2. Largest Lyapunov Exponent Analysis

To further characterize the nonlinear behavior of the delayed network model, we compute the largest Lyapunov exponent (LLE) as a function of the coupling strength β for fixed delay values τ=1,…,10. The largest Lyapunov exponents (LLEs) are computed numerically from time series using the standard algorithm based on tangent space evolution. After discarding an initial transient of $N_{0}$ iterations, the exponent is estimated over N iterations. All simulations were performed with a transient length of 5000 iterations and a total iterations equal to 20,000 iterations using our own Python code. The LLE provides a quantitative measure of sensitivity to initial conditions and allows classification of the dynamical regimes:

(41) LLE<0⇒asymptoticstability,LLE=0⇒bifurcationthreshold,

(42) LLE>0⇒chaoticdynamics.

**Figure 4:**
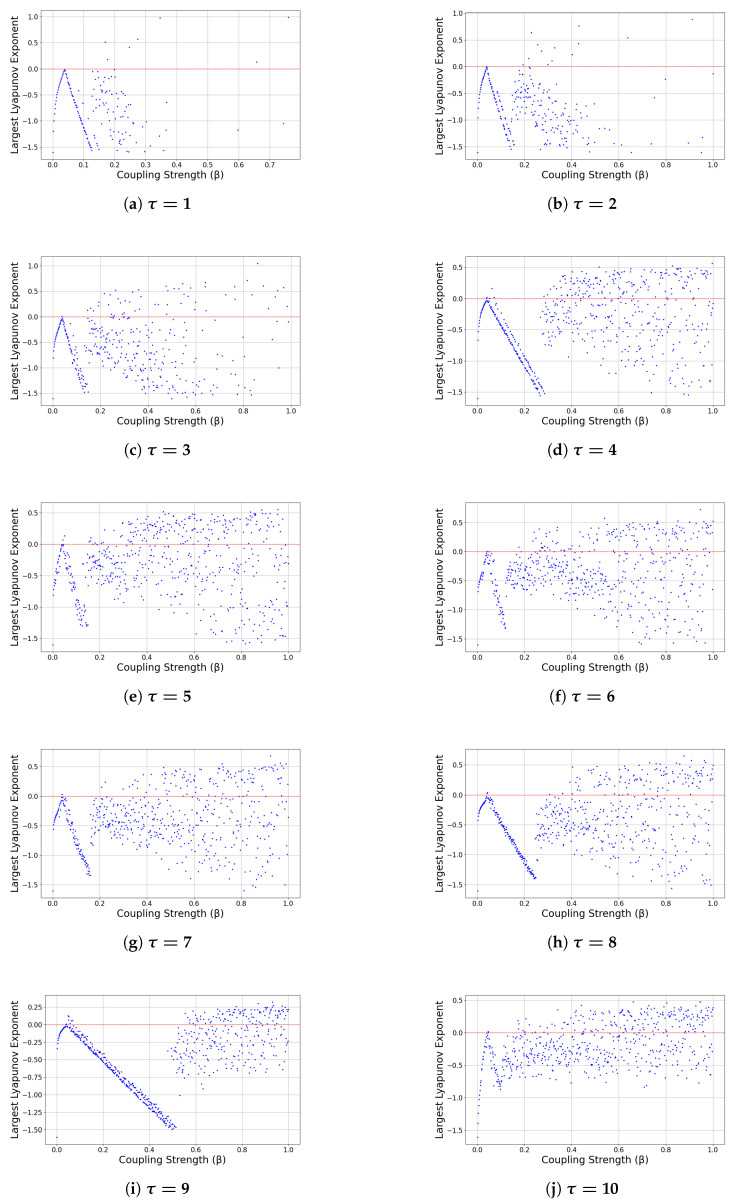
Largest Lyapunov exponent (LLE) as a function of the forgetting parameter β for increasing delay values τ=1,…,10. Positive LLE values indicate chaotic dynamics, while negative values correspond to stable asymptotic behavior.

Figure 4a–j displays the dependence of the LLE on β for increasing delay.

(i) Small coupling regime

For sufficiently small values of β, the LLE is strongly negative for all delays (approximately between −1.5 and −1). This confirms global contraction of trajectories and validates the stability results obtained from the Lyapunov–Krasovskii functional approach. In this regime, the trivial equilibrium remains globally attracting.

(ii) Transition to instability

As β increases, the LLE approaches zero and crosses into positive values at delay-dependent critical thresholds. This crossing corresponds to the loss of stability observed in the bifurcation diagrams and signals the onset of complex dynamics. For small delays (τ=1,2), the positive-LLE region is narrow, indicating limited chaotic behavior; however, for moderate delays (τ=3,4,5) the zero-crossing occurs earlier and the chaotic interval becomes wider.

As α increases, the system transitions from negative LLE (stable regime) to near-zero values (bifurcation threshold), then finally to positive LLE (chaotic regime). This confirms that α acts as a primary control parameter governing instability onset.

(iii) Delay-induced chaos

For larger delays (τ≥4), broad intervals of β exhibit positive LLE values (see Figure 4d–j). The persistence of positive exponents over extended parameter ranges indicates deterministic chaos and strong sensitivity to initial conditions. The width of the chaotic region increases monotonically with the delay, demonstrating that delay acts as a primary destabilizing mechanism. This behavior is consistent with classical results for delayed dynamical systems, where increasing memory effectively raises the system dimension and promotes stretching–folding dynamics.

(iv) Strong coupling regime

For large β, the LLE often remains positive, particularly for larger delays. Although the bifurcation diagrams suggest saturation of trajectories near ±1, the LLE plots reveal that chaotic fluctuations may persist in this regime. Thus, nonlinear saturation of the **tanh** function does not fully suppress instability when significant delay is present.

### 5.3. Reconstruction of the Attractor and Dimension Analysis

To investigate the intrinsic dimensionality and nonlinear structure of the dynamics generated by the networked delayed system, we employ standard tools of nonlinear time series analysis, namely, the false nearest neighbors (FNN) method, the Grassberger–Procaccia algorithm for correlation dimension estimation, and delay-coordinate reconstruction of the attractor.

#### 5.3.1. Embedding Dimension via False Nearest Neighbors

The first step is to determine an appropriate embedding dimension m for reconstructing the phase space from a scalar time series $x_{i}\left(t\right)$. This is achieved using the false nearest neighbors method. The idea is that if the embedding dimension is too low, then points appearing close in the reconstructed space are actually far apart in the true phase space. As the embedding dimension increases, the fraction of such “false” neighbors decreases.

**Figure 5:**
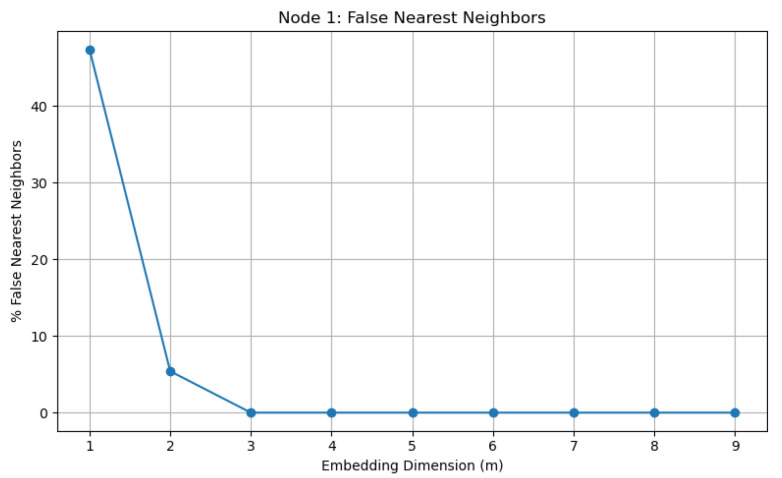
Percentage of false nearest neighbors as a function of embedding dimension for node i=1.

The results for node i=1 are shown in Figure 5. A sharp decrease in the percentage of false nearest neighbors can be observed from approximately 45% at m=1 to nearly zero at m=3. For m≥3, the percentage remains essentially zero. This indicates that an embedding dimension m=3 is sufficient to unfold the attractor without projection-induced overlaps. Consequently, the effective dimension of the underlying dynamics is low.

#### 5.3.2. Correlation Dimension

To further quantify the geometric complexity of the attractor, we compute the correlation sum

(43) $$C\left(r\right)=\frac{1}{N^{2}}\sum_{i,j}1\{∥X_{i}-X_{j}∥<r\},$$

where $X_{i}$ denotes the delay-embedded vectors. In the scaling region, the correlation sum behaves as

(44) $$C\left(r\right)∼r^{D_{2}},$$

where $D_{2}$ is the correlation dimension.

**Figure 6:**
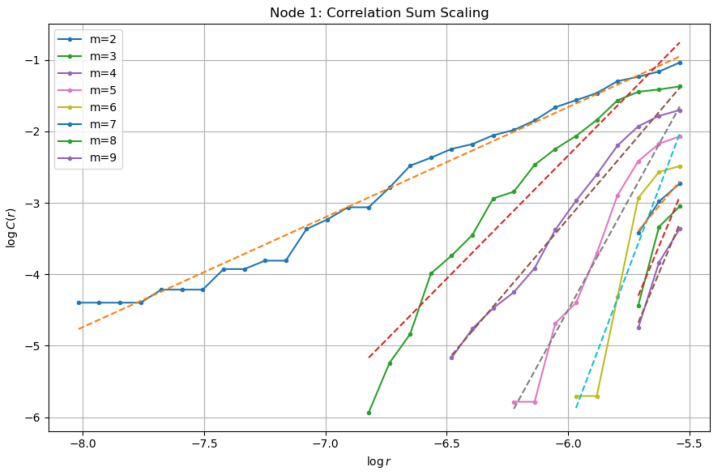
Log–log scaling of the correlation sum for different embedding dimensions.

Figure 6 presents the dependence of logC(r) on logr for several embedding dimensions. For moderate values of r, an approximately linear scaling region is observed in which the slope provides an estimate of $D_{2}$.

**Figure 7:**
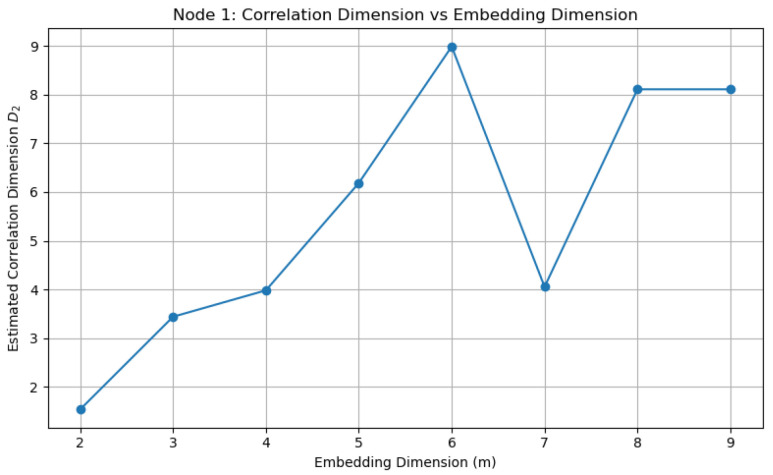
Estimated correlation dimension $D_{2}$ as a function of embedding dimension m.

The resulting estimates of the correlation dimension as a function of m are shown in Figure 7. Although the estimates initially increase with m, they exhibit significant fluctuations and do not clearly stabilize within the tested range. This behavior suggests that the attractor is not strictly low-dimensional in the classical deterministic sense but rather possesses a more complex structure, possibly influenced by the network interactions and stochastic topology.

#### 5.3.3. Reconstructed Attractor

Using the embedding dimension m=3 suggested by the FNN analysis, we reconstruct the attractor in three-dimensional space:

(45) $$X\left(t\right)=\left(x(t),x(t+\tau ),x(t+2\tau )\right).$$

**Figure 8:**
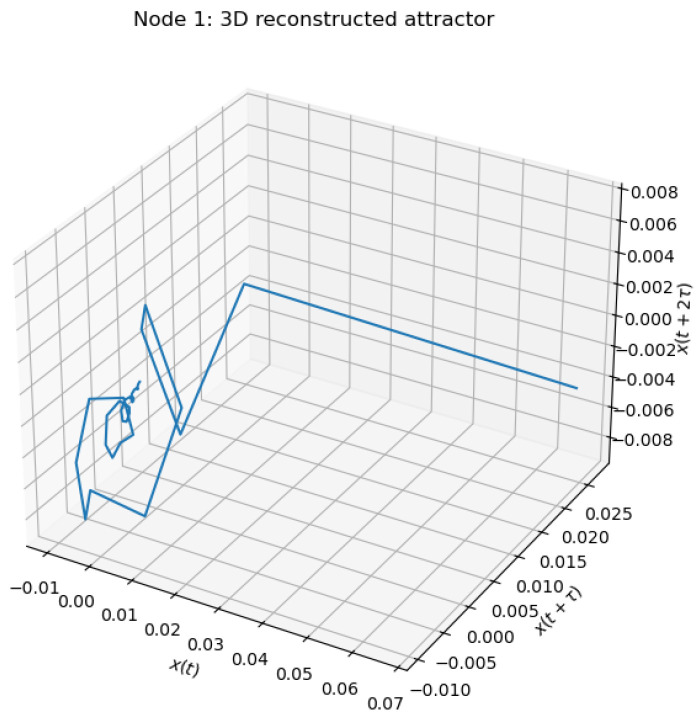
Three-dimensional delay-coordinate reconstruction of the attractor for node i=1.

The reconstructed attractor is depicted in Figure 8.

The geometry of the attractor reveals a strongly contracting structure, with trajectories rapidly approaching a narrow region of the phase space. The absence of a richly folded structure and the presence of near-linear segments indicate that, for the chosen parameter regime, the system exhibits predominantly dissipative dynamics with limited complexity. This observation is consistent with the small coupling strength and relatively strong damping present in the model.

Combining the above results, we can conclude that the system operates in a regime characterized by low effective dimensionality and strong contraction. The rapid decay of false nearest neighbors indicates that a small embedding dimension is sufficient, while the correlation dimension analysis suggests that the attractor does not exhibit clear fractal scaling within the examined range. The reconstructed attractor further supports this conclusion, displaying a simple geometric structure without pronounced chaotic folding.

These findings are consistent with the stability properties established earlier via Lyapunov methods, indicating that in this parameter regime the networked delayed system does not generate high-dimensional chaos, but rather converges towards a structured low-complexity attractor.

## 6. Unified Discussion: Parameter-Induced Complexity in the Delayed Network Model

The numerical investigations performed with respect to the coupling strength β and the attention–decay parameter α reveal a coherent and structurally rich scenario of parameter-induced complexity in the delayed Erdős–Rényi network model. Although β and α affect different mechanisms in the dynamics (network interaction vs. self-retention), their bifurcation structures exhibit strong qualitative similarities, especially in the presence of discrete delays.

### 6.1. Role of the Coupling Strength β

When β is small, the delayed interaction term is weak and the system is dominated by local self-dynamics. In this regime, the network rapidly converges to stable equilibria or low-period oscillations, depending on the delay value. As β increases, the influence of delayed neighbors becomes stronger and a transition from stable behavior to complex oscillatory regimes is observed.

The bifurcation diagrams with respect to β demonstrate a classical route to complexity: first, branching of equilibria; next, the appearance of multi-periodic behavior; and finally, dense irregular regions indicating chaos-like dynamics. The critical coupling threshold at which this transition occurs decreases as the delay τ increases. Thus, larger delays reduce the stability margin and enhance sensitivity to coupling intensity.

This observation is further supported by the largest Lyapunov exponent (LLE) analysis. For small β, the LLE remains negative, confirming exponential convergence. As β increases, the LLE approaches zero and eventually becomes positive in extended parameter intervals (particularly for larger τ), indicating the onset of chaotic dynamics. The widening of positive-LLE regions with increasing delay confirms that delayed feedback acts as a complexity amplifier.

### 6.2. Role of the Attention–Decay Parameter α

The α parameter controls the instantaneous self-feedback through the factor (1−α). For α≪1, the self-retention is strong and the system exhibits polarized stable states near ±1, reflecting saturation of the nonlinear activation function.

As α approaches **1**, the effective instantaneous feedback weakens. Near α≈1, the system dynamics become dominated by delayed network interactions, producing a broad transition region characterized by dense and vertically-spread asymptotic values in the bifurcation diagrams. This regime corresponds to multistability, quasi-periodicity, and chaotic behavior.

For α>1, the instantaneous feedback changes its sign, introducing an additional destabilizing mechanism. Beyond the transition region, the system reorganizes into large-amplitude states, but the irregular region remains wide and structured for sufficiently large delays. As in the analysis of β, increasing the delay enlarges the parameter interval associated with the complex dynamics.

### 6.3. Common Structural Mechanism

Despite their different interpretations, β and α influence a common structural balance:

(46) instantaneousself-dynamicsvs.delayednetworkfeedback.

Complexity emerges whenever this balance is sufficiently perturbed. In particular:

- Small β or small α (strong self-damping dominance) ⇒ stable equilibria.
- Intermediate values (competition between mechanisms) ⇒ bifurcations and multistability.
- Large β or α≈1 (delay-dominated regime) ⇒ chaotic or high-dimensional oscillatory behavior.
- Increasing delay τ systematically lowers stability thresholds and broadens chaotic regions.

Hence, the delay acts as a universal destabilizing factor that magnifies the nonlinear effects induced by both the coupling and self-dynamics parameters.

### 6.4. Delay as an Amplifier of Complexity

Across all numerical experiments, the delay τ plays a decisive role. As τ increases, the following effects are observed:

1. Onset of instability occurs for smaller variations of β or α.
2. The bifurcation diagrams become denser and more irregular.
3. Positive LLEs appear over wider parameter intervals.
4. Multistability and sensitivity to initial conditions become more pronounced.

This behavior is consistent with the well-known fact that delay effectively increases the dimension of the phase space, enabling high-dimensional attractors and chaotic regimes.

### 6.5. Global Dynamical Scenario

The bifurcation and Lyapunov analyses together reveal a coherent picture of how the system evolves as its parameters vary. Rather than a collection of isolated regimes, the dynamics follow a clear progression from simple to increasingly complex behavior:

(47) Stableequilibrium⟶symmetrybreaking

(48) ⟶oscillatorydynamics⟶complexorchaoticbehavior.

The coupling strength β primarily governs this transition. Weak coupling suppresses information propagation, leading to a stable inactive state. As the interaction strength increases, the system departs from this trivial regime and develops polarized states, reflecting competing influences within the network. Further increases in β lead to irregular and fluctuating dynamics, and eventually to strongly nonlinear regimes characterized by large-amplitude or chaotic behavior. In this sense, β controls how strongly local interactions translate into collective dynamics.

The largest Lyapunov exponent provides a complementary viewpoint that is more global. Its sign distinguishes qualitatively different regimes: negative values indicate stable convergence, values near zero mark the onset of instability, and positive values correspond to sensitive chaotic evolution. From this perspective, the transition towards complex dynamics can be interpreted as a gradual loss of predictability in which small perturbations increasingly influence long-term behavior.

The α parameter representing attention and decay effects modulates the temporal persistence of information. Small values favor long memory and stable configurations that are often polarized, while values near unity introduce a balance between reinforcement and forgetting that promotes variability and irregular dynamics. Larger values of α again reshape the dynamics, typically producing pronounced but more structured responses. Thus, α acts as a tuning parameter for the system’s temporal coherence.

Across all parameter settings, the delay τ emerges as the key factor driving complexity. Increasing the delay consistently enlarges the parameter regions in which oscillations and chaotic behavior occur. This reflects the role of delay as a source of memory and feedback, effectively increasing the system’s dynamical dimension and enabling richer temporal patterns.

Overall, the model exhibits a transition from stable or weakly polarized states, through oscillatory and branching behavior, to high-dimensional chaotic dynamics. These regimes are not isolated, but rather arise from the interplay between interaction strength, memory decay, and delay. The bifurcation diagrams capture these transitions visually while the Lyapunov exponents provide a quantitative measure of their stability, together offering a comprehensive view of complex information spreading in delayed networks.

In conclusion, the delayed network model exhibits a rich spectrum of nonlinear phenomena similar to classical delayed dynamical systems, but with additional structural complexity arising from random network topology. Our combined bifurcation and Lyapunov analyses demonstrate that parameter-induced complexity is not an artifact of deterministic low-dimensional models; instead, it persists and is even amplified in high-dimensional stochastic network settings.

## 7. Conclusions

This paper investigates a delayed network model of information spreading, focusing on the interplay between interaction strength, memory decay, and time delay in shaping the system’s dynamics. Through a combination of bifurcation analysis and Lyapunov exponent computation, we show that the model exhibits a rich spectrum of behaviors ranging from stable equilibria and polarized states to oscillatory regimes and high-dimensional chaotic dynamics. In particular, results demonstrate that time delay plays a central role in amplifying nonlinear effects and promoting the emergence of complex temporal patterns.

The main contributions of this work are threefold: first, we propose a discrete-time network model that incorporates both delayed interactions and nonlinear response functions, providing a flexible framework for studying information spreading processes; second, we perform a systematic numerical analysis of the model, revealing how key parameters govern transitions between qualitatively distinct dynamical regimes; and third, we combine bifurcation diagrams with Lyapunov exponent analysis to obtain both qualitative and quantitative insight into the stability and unpredictability of the system.

Despite these contributions, several limitations should be noted. The network structure is based on the Erdős–Rényi model, which does not capture important features of real social networks such as clustering, community structure, or heterogeneous degree distributions. In addition, the analysis is primarily numerical, and the theoretical characterization of stability regions remains conservative. Furthermore, the choice of activation function and model parameters, while motivated by analytical convenience, may influence the quantitative behavior of the system.

These limitations suggest several directions for future research. An important extension would be to consider more realistic network topologies, including small-world and scale-free networks, which are better able to capture empirical social structures. Another promising direction is the development of sharper analytical tools for stability and bifurcation analysis, which could reduce the conservatism of the current estimates. It would also be of interest to investigate alternative activation functions and adaptive mechanisms, as well as to incorporate data-driven calibration and validation using real-world information spreading datasets. Finally, extending the framework to heterogeneous delays or stochastic interactions could further enhance its applicability to complex social systems.

Another promising direction is the investigation of richer nonlinear mechanisms, including multistability, fractional-order effects, and adaptive memory structures, which have recently been shown to generate hyperchaotic behavior in discrete dynamical systems and memristive neural maps [16,17]. Such extensions may provide additional insight into the coexistence of multiple spreading regimes and complex attractor structures in delayed information networks.

Overall, the results highlight the importance of delayed interactions in shaping collective dynamics and provide a foundation for further studies on nonlinear information spreading in complex networks.

## Data Availability

The original data presented in the study are openly available in TransLeader platform at [https://tl.ubb.edu.pl/en/progress/new-scientific-publication-within-the-transleader-project-10.html] (accessed on 23 March 2026).

## Abbreviations

The following abbreviations are used in this manuscript:

RNN	Recurrent Neural Network
LLE	Largest Lyapunov Exponent
LMI	Linear Matrix Inequality
FNN	False Nearest Neighbors
